# Cardanol isolated from Thai Apis mellifera propolis induces cell cycle arrest and apoptosis of BT-474 breast cancer cells via p21 upregulation

**DOI:** 10.1186/s40199-015-0138-1

**Published:** 2015-12-22

**Authors:** Sureerat Buahorm, Songchan Puthong, Tanapat Palaga, Kriengsak Lirdprapamongkol, Preecha Phuwapraisirisan, Jisnuson Svasti, Chanpen Chanchao

**Affiliations:** Program in Biotechnology, Faculty of Science, Chulalongkorn University, 254 Phayathai Road, Bangkok, 10330 Thailand; Institute of Biotechnology and Genetic Engineering, Chulalongkorn University, 254 Phayathai Road, Bangkok, 10330 Thailand; Department of Microbiology, Faculty of Science, Chulalongkorn University, 254 Phayathai Road, Bangkok, 10330 Thailand; Laboratory of Biochemistry, Chulabhorn Research Institute, Vipawadee Rangsit Highway, Bangkok, 10210 Thailand; Department of Chemistry, Faculty of Science, Chulalongkorn University, 254 Phayathai Road, Bangkok, 10330 Thailand; Department of Biology, Faculty of Science, Chulalongkorn University, 254 Phayathai Road, Bangkok, 10330 Thailand

**Keywords:** *Apis mellifera*, Cardanol, Cell arrest, Cell death, p21, Propolis

## Abstract

**Background:**

Cardanol was previously reported to be an antiproliferative compound purified from Thai *Apis mellifera* propolis. By morphology, it could induce the cell death to many cancer cell lines but not the control (non-transformed human foreskin fibroblast cell line, Hs27). Here, it was aimed to evaluate the molecular effects of cardanol on breast cancer derived cell line (BT-474).

**Methods:**

Morphological changes in BT-474 cells induced by cardanol compared to doxorubicin were evaluated by light microscopy, cytotoxicity by using the 3- (4, 5-dimethyl-thiazol-2-yl) 2, 5-diphenyl-tetrazolium bromide (MTT) assay, induction of cell cycle arrest and cell death by flow cytometric analysis of propidium iodide and annexin-V stained cells, and changes in the expression level of genes involved in the control of apoptosis and the cell cycle by quantitative reverse transcriptase-PCR (qRT-PCR) and western blot analyses.

**Results:**

It revealed that cardanol induced a time- and dose-dependent cytotoxicity along with cell shrinkage and detachment from substratum. Cardanol caused cell cycle arrest at the G_1_ subphase (as opposed to at the G_2_/M subphase seen with doxorubicin) and cell death by late apoptosis, with both late apoptosis (27.2 ± 1.1 %) and necrosis (25.4 ± 1.4 %) being found in cardanol treated cells after 72 h, compared to a lower proportion of apoptosis (4.3 ± 0.4 %) and higher proportion of necrosis (35.8 ± 13.0 %) induced by doxorubicin. Moreover, cardanol changed the transcript expression levels of genes involved in the control of apoptosis (increased *DR5* and *Bcl-2* expression and decreased *Mcl-1*, *MADD* and *c-FLIPP*) and cell division (increased p21 and E2FI and decreased cyclin D1, cyclin E, CDK4 and CDK2 expression), as well as increasing the level of p21 p*-ERK*, p-JNK and p-p38 and decreasing cyclin D. This accounts for the failure to progress from the G_1_ to the S subphase.

**Conclusion:**

Cardanol is a potential chemotherapeutic agent for breast cancer.

**Electronic supplementary material:**

The online version of this article (doi:10.1186/s40199-015-0138-1) contains supplementary material, which is available to authorized users.

## Background

Propolis, one of honeybee products, has mostly been used in traditional medicine. As known, the components and properties of propolis mainly depend on the geographic location and bee species. For example, the propolis derived from bees foraging on poplar (*Populus nigr*a L.) was found to consist of plant resin (50 %), wax (30 %), oil (10 %), pollen (5 %) and other components (5 %) [1].

Propolis has been reported to show many bioactivities, including antibacterial, antiviral, anti-inflammatory and antioxidant activities [2–5]. These reports have typically presented the potential of propolis in the form of either crude or purified extracts/compounds and assayed in either an in vitro or an in vivo model. Also, it was linked to the regulation of gene expression in many types of cells like macrophages, spleenocyte cells and human monocytes [6, 7].

Interestingly, propoelix™, a water-soluble extract of propolis, has been used successfully in the treatment of patients with dengue hemorrhagic fever [8]. In addition, propolis has been reported to be a very rich source of polyphenolic compounds, flavonoids and fatty acids [9]. For example, baccharin isolated from Brazillian propolis and its analogs were able to inhibit aldo-keto reductase 1C3 (AKR1C3), which is involved in castration resistant prostate cancer [10]. The administration of caffeic acid phenethyl ester (CAPE) at 5 μM/kg in mice by intraperitoneal injection showed anti-depressant activity in mice receiving chronic unpredictable stress for 21 consecutive days. Downregulation of p38MAPK phosphorylation by CAPE, which contributed to enhance glucocorticoid receptor function, has also been reported [11].

The molecular mechanism of those active compounds has been revealed, or at least in part. For example, CAPE (25 μM) induces apoptosis in the HeLa cervical cancer cell line (ME 180) and induces cell cycle arrest at the S and G_2_/M subphases. The expression level of the E2F-1 target gene, cyclin A, cyclin E, apoptosis protease activating of factor-1 (Apaf-1) and myeloid leukemia cell differentiation protein (Mcl-1) were upregulated but cyclin B was down-regulated [12].

In addition, chrysin significantly reduced the serum levels of the pro-inflammatory cytokines IL-1β and IL-6 in high fat diet/streptozotocin -induced type 2 diabetic rats. Since these pro-inflammatory cytokines, along with especially TNF-α, have an important function in insulin resistance and inflammatory responses, chrysin could be a new target for the treatment of type 2 diabetes [13].

Cardanol, a phenolic compound found in members of the cashew tree (Anacardiaceae) family, has been associated with diverse biological effects, such as antiproliferative, antimicrobial and antioxidant activities [14–17]. However, the molecular mode of action of cardanol is unknown. In this research, the BT-474 cell line, as an in vitro breast cancer model, was focused because it is the leading cause of death in Thai women [18]. Here, the induction of cell cycle arrest as well as program cell death was reported. The change in the expression level of genes that control these functions was also investigated. Finally, a molecular mechanism of cardanol action on the BT-474 cell line is proposed.

## Methods

### Preparation of propolis

Propolis from *Apis mellifera* was collected from the hives at a bee farm in Pua district, Nan province, Thailand in January, 2012. It was wrapped in aluminum foil and kept in the dark at −20 °C until used. The extraction and enrichment to apparent homogeneity of cardanol from the propolis, along with the one-dimensional thin layer chromatography (1D-TLC), was performed as previously reported [14].

### Cell culture

The BT-474 cells (ATCC no. HTB 20) was cultured in complete medium (CM) comprised of Roswell Park Memorial Institute (RPMI) 1640 medium containing 5 % (v/v) fetal calf serum. Cells were seeded at 1 × 10^5^ cells/5 ml CM/ 25-cm^2^ flask and incubated at 37 °C with 5 % (v/v) CO_2_. Cells were re-passaged when they reached 70–80 % confluency.

### Cytotoxicity

Cytotoxicity was evaluated indirectly from MTT assay. Thus, the results are influenced by changes in the average cell proliferation rate and/or cell viability, and the reduction in the total number of viable cells is herein referred to as the cytotoxicity without delineation of these two components. BT-474 cells (5 10^3^ cells in 198 μl) were seeded in each well of a 96 well plate, and incubated at 37 °C with 5 % (v/v) CO_2_ for 24 h. Then 2 μl of cardanol or doxorubicin, dissolved in dimethylsulfoxide (DMSO) to a concentration of 10000, 1000, 100, 10, 1 and 0.1 μg/ml for cardanol and 50 μg/ml for doxorubicin, was added to the wells in triplicate, along with DMSO only (2 μl/well) as the solvent (no treatment) control. The cells were then incubated for 72 h before 10 μl of 5 mg/ml of MTT solution was added to each well and incubated for another 4 h. After that, the media was removed and replaced with 150 μl of DMSO and 25 μl of 0.1 M glycine and gently aspirated to lyse the cells and dissolve the formazan crystals. The absorbance was then measured at 540 nm (A_540_) by a microplate reader. Setting the total number of viable cells in the control culture to be 100 %, the relative percentage of viable cells was calculated from Eq. (1):

1 $$ \mathrm{Relative}\ \mathrm{number}\ \mathrm{of}\ \mathrm{viable}\ \mathrm{cells} = \left({\mathrm{A}}_{540}\mathrm{of}\ \mathrm{sample}\ /\ {\mathrm{A}}_{540}\mathrm{of}\ \mathrm{control}\right) \times 100 $$

The concentration of the test compound that caused a 50 % maximal inhibition of the viable cell number (IC_50_) was derived from the graphical plot of the relative number of viable cells *vs.* test compound concentration.

### Growth curve of BT-474 cells

BT-474 cells treated with solvent only (control) or with cardanol at the IC_50_ value (15.6 ± 1.76 μg/ml) were assayed for the relative number of viable cells using the MTT assay after 1, 2, 3, 5 and 7 d of culture. The graph of relative number of viable cells *vs.* time was drawn, where the trend line was compared to the control cell line.

### Cell morphology

BT-474 cells (2 × 10^5^ cells/ml) were cultured in CM with the addition of (i) the DMSO solvent only (Control), (ii) 30 μg/ml of cardanol and (iii) 0.5 μg/ml of doxorubicin (positive control). The morphology of the cells was observed after 0, 24, 48, 72 and 96 h incubation using inverted light microscope (Ziess, Jena) connected to a digital camera (Canon EOS 7D, Tokyo).

### Detection of apoptosis and necrosis

BT-474 cells (3–5 × 10^6^ cells/ml) were cultured in CM with the addition of (i) the DMSO solvent only (Control), (ii) 30 μg/ml of cardanol and (iii) 0.5 μg/ml of doxorubicin (positive control). After the indicated time in culture (24–72 h) the cells were harvested by centrifugation (3000 × g, 4 °C for 10 min), washed in 1 ml of cold 1 x phosphate buffer saline (PBS) and harvested as before. The pellet was resuspended in 50 μl of 1 × binding buffer pH 7.4 (10 mM Hepes, 140 mM NaCl and 2.5 mM CaCl_2_) and stained with the addition 1 μl of annexin V (Alexa Fluor 488 conjugate, Life Technologies, Carlsbad, CA) and 5 μl of 1 mg/ml propidium iodide (PI) solution (Sigma Aldrich, St. Louis, MO) in the dark at room temperature (RT) for 30 min. Cells were then analyzed by flow cytometry on a FC 500 MPL cytometer (Beckman Coulter, Brea, CA) recording 2 events (cells).

### Detection of cell cycle arrest

BT-474 cells (1–100 × 10^6^ cells/ml) were cultured in CM with the addition of (i) the DMSO solvent only (Control), (ii) 30 μg/ml of cardanol and (iii) 0.5 μg/ml of doxorubicin (positive control) for 24, 48 and 72 h and then harvested and washed as above. The cell pellet was resuspended and fixed in 500 μl of cold PBS and 200 μl of 70 % (v/v) ethanol at −20 °C overnight or on ice for 4 h. Cells were then harvested and washed as above and the pellet resuspended in 250 μl of PBS with 0.1 mg/ml RNAse and incubated at 37 °C for 30 min. After harvesting, the cells were resuspended in 12.5 μl of 1 mg/ml PI and incubated at RT in the dark for 30 min before being analyzed by flow cytometry on a FC 500 MPL cytometer (Beckman Coulter) recording 2 events per sample. The obtained linear fluorescence profile was interpreted in terms of the (1) sub G_1_ phase (apoptotic cells), (ii) G_1_ phase (diploid chromosome content), (iii) S phase (DNA synthesis) and (iv) G_2_/M subphase (double diploid).

### Change in gene expression levels

#### Transcript expression levels

BT474 cells were cultured in CM with the addition of (i) the DMSO solvent only (Control), (ii) 30 μg/ml of cardanol and (iii) 0.5 μg/ml of doxorubicin (positive control) for 72 h, and then harvested. Total RNA was then extracted from them using the RNeasy Plus Mini Kit (Qiagen, Hilden) as per the suppliers protocol. The extracted RNA was eluted in 20 μl of RNase-free H_2_O and the absorbance at 260 and 280 nm (A_260_ and A_280_, respectively) was measured. The concentration of RNA was calculated from Eq. (2),

2 $$ \mathrm{Concentration}\ \mathrm{of}\ \mathrm{R}\mathrm{N}\mathrm{A}\ \left(\upmu \mathrm{g}/\mathrm{ml}\right) = \left({\mathrm{A}}_{260}\right) \times \mathrm{dilution}\ \mathrm{factor} \times (40). $$

The purity of the extracted RNA was estimated from the A_260_/A_280_ ratio. The RNA samples were stored at −20 °C until use.

The transcript expression levels of the selected genes were then assayed by single-stage qRT-PCR. Two groups of genes were selected for screening. The first group were the death receptor group of the apoptosis regulated genes *b-cell lymphoma-2* (*Bcl-2*), *Mcl-1*, *mitogen activating protein-kinase activating death domain* (*MADD*), *cellular FLICE-like inhibitory protein* (*c-FLIP*) and *human death receptor 5* (*DR5*). The second group were the cell cycle regulating genes of *p21*, *cyclin D1*, *cyclin E*, *cyclin A*, *cyclin-dependent kinase 4* (*CDK4*), *CDK6* and *CDK2*.

The reaction mixture was prepared using the One Step SYBR PrimeScript RT-PCR Kit II (Takara, Tokyo) as per the manufacturer’s protocol. Each qRT-PCR reaction mixture (20 μl final volume) contained total RNA (10 ng), 10 μl of 2x one step SYBR RT-PCR buffer, 1 μl of Prime Script 1 step enzyme mix, 0.5 μl of each gene fragment specific forward and reverse PCR primer (20 μM stock) and RNase-free d-H_2_O. The respective forward and reverse primers are listed in Table 1. The PCR thermocycling was performed at 95 °C for 15 min, followed by 40 cycles of 94 °C for 15 s, x °C for 30 s and 72 °C for 30 s, where x is gene specific and is given in Table 1. The relative expression level of each gene was normalized to the expression level of the ß-actin gene as an internal control. The crossing point (Cp) was used to calculated the relative gene expression level as per Eq. (3),

**Table 1:** Forward and reverse primers (5’ → 3’) used in the qRT-PCR

Gene	Nucleotide sequence of F primer	Nucleotide sequence of R primer	Annealing temp. (°C)	Reference
*ß-actin*	GACCTGACTGACTACCTCATGA	AGCATTTGCGGTGGACGATGGAG	55	Lirdprapamongkol et al. [20]
*MADD*	TCAACCCACTCATCTATGGCAATG	GCGGAATTGAAGAACCGTACCA	60	Li et al. [36]
*c-FLIP*	CCAGAGTGTGTATGGTGTGGAT	TCTCCCATGAACATCCTCCTGAT	60	Li et al. [36]
*Bcl-2*	TGGGATGCGGGAGATGTG	CGGGATGCGGCTGGAT	60	Li et al. [36]
*Mcl-1*	AGCAGAGGAGGAGGAGGAC	GCCTGCTCCCGAAGGTA	55	Lirdprapamongkol et al. [20]
*DR5*	TGCTGCTCAAGTGGCGC	GGCATCCAGCAGATGGTTG	60	Pillai et al. [37]
*P21*	CACTCCAAACGCCGGCTGATCTTC	TGTAGAGCGGGCCTTTGAGGCCCTC	55	Weglarz et al. [38]
*E2F1*	GCCACTGACTCTGCCACCA	GGACAACAGCGGTTCTTGCT	60	Galanti et al. [39]
*Cyclin A*	GAAGACGAGACGGGTTGCA	AGGAGGAACGGTGACATGCT	60	Galanti et al. [39]
*Cyclin D1*	AATGACCCCGCACGATTTC	TCAGGTTCAGGCCTTGCAC	60	Ullmannova et al. [40]
*Cyclin E*	TTCTTGAGCAACACCCTCTTCTGCAGCC	TCGCCATATACCGGTCAAAGAAATCTTGTGCC	58	Potemski et al. [41]
*CDK2*	TTTGGAGTCCCTGTTCGTAC	TGCGATAACAAGCTCCGTCC	58	Chiang et al. [42]
*CDK4*	CTTTGACCTGATTGGGCTGC	GGAGAGGTGGGAGGGGAATG	58	Chiang et al. [42]
*CDK6*	TCTTGCTCCAGTCCAGCTAC	AGCAATCCTCCACAGCTCTG	60	Ullmannova et al. [40]

3 $$ \mathrm{Relative}\ \mathrm{expression}\ \mathrm{level} = {2}^{\left(\mathrm{C}\mathrm{p}\ \mathrm{actin}-\mathrm{C}\mathrm{p}\ \mathrm{target}\right)}. $$

The Cp value is correlated to the amount of the initial template and so indicates the expression of the target mRNA [19].

#### Protein expression levels

Changes in the expression level of selected proteins were evaluated by western blot analysis following the protocol of Lirdprapamongkol et al. [20] with slight modification. BT-474 cancer cells (2 × 10^5^ cells/ml) were cultured in CM with the addition of (i) the DMSO solvent only (Control), (ii) cardanol at the 2x IC_50_ concentration (30 μg/ml) and (iii) 0.5 μg/ml of doxorubicin (positive control) for 24 h. Cells were then harvested and lysed in 150 μl of radioimmunoprecipitation assay buffer, which contained 1x halt protease phosphatase and phosphatase inhibitor cocktail with EDTA (Thermo Scientific, Waltham, MA), on ice. The concentration of protein in the lysate was measured by the Bradford assay.

Twenty μg of protein was loaded per well of a sodium dodecyl sulfate polyacrylamide gel (SDS-PAGE) with a 7 and 4 % (w/v) acrylamide separating and stacking gel, respectively. After electrophoresis at 15 mA for 105 min, the protein was transferred to immobilon-P nylon membrane (Millipore, Billerica, MA) by electroblotting at 100 V for 90 min. The membrane was later blocked with 3 % (w/v) bovine serum albumin (BSA) for 1 h with gentle shaking at RT. After that, the membrane was cut and probed with the primary antibodies (Cell Signaling Technology, Danvers, MA) diluted in 3 % (w/v) BSA to 1: 1000 (all except for anti-pERK that was 1: 5000) overnight at 4 °C in the dark. The membrane was washed in 1x TBS/T pH 7.6 (20 mM Tris and 137 mM NaCl) and incubated with the diluted horseradish peroxidase-conjugated secondary antibody (Promega, Fitchburg, WI) in TBS/T containing 5 % (w/v) skim milk (1: 10000 mouse, 1: 5000 rabbit) with gentle shaking at RT for 1 h. The bound secondary antibodies were then visualized using western bright ECL reagents (Advansta, Menlo Park, CA) as per the supplier’s protocol and the image was captured using an Image Quant LAS 4000 mini instrument (GE Healthcare Life Sciences, Little Chalfont).

### Statistical analysis

Data are expressed as the mean ± one standard deviation (1 S.D.), derived from triplicate replications in each experiment. The data were analyzed by one way analysis of variance (ANOVA) followed by Tukey’s test of multiple comparisons to test for the significance of differences in the means. Significance was accepted at the *p* < 0.05 level. All analyses were performed using the SPSS program version 19.0.

## Results

### Cardanol isolation

**Fig. 1:**
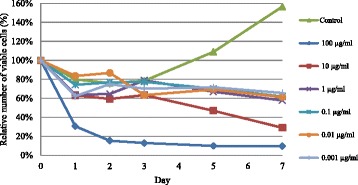
Growth curve of the solvent only (Control) and cardanol treated (0.001–199 μg/ml) BT-474 cell line. Data are shown as the mean ± 1SD, derived from three independent repeats

Starting with 90 g of *Apis mellifera* propolis, 1.54 g of crude dichloromethane extract (CDE) was obtained and then further fractionated by successive quick column and adsorption column chromatography (CC). The obtained cardanol (0.52 mg) was confirmed from its 1D-TLC derived R_f_ value [14] and from its mass spectrometry derived spectrum. The IC_50_ value for the cytotoxicity against BT-474 cells was calculated to be 15.6 ± 1.76 μg/ml (Fig. 1), which was close to the 14.0 ± 1.0 μg/ml previously reported on this cell line [14].

In order to confirm that the BT-474 cells were healthy under these culture conditions and were responsive to cardanol, the growth of BT-474 cells in CM with either DMSO only (solvent control) or with various concentrations of cardanol (0.001–100 μg/ml) was evaluated, and is shown in terms of relative to the initial amount as 100 % (Fig. 1). Among the four phases of a usual growth curve, the total number of viable cells was recorded from the lag phase to the log (exponential growth) phase because treated cells at any cardanol concentration started to die at the end of the log phase. Overall, the inhibition by cardanol was time- and dose-dependent manner.

### Morphological changes in BT-474 cells

**Fig. 2:**
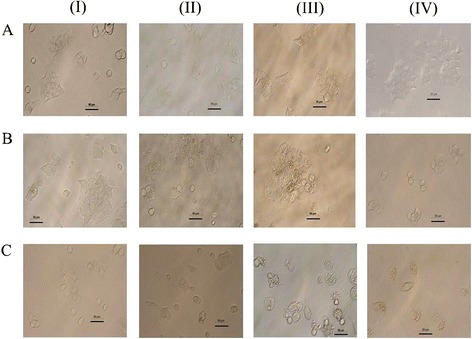
Morphology of the **a** control BT-474 cells and those treated with **b** cardanol at the IC_50_ concentration (15.6 ± 1.76 μg/ml) and **c** doxorubicin (0.5 μg/ml) for (I) 24 h, (II) 48 h, (III) 72 h and (IV) 96 h of incubation. The scale bar represents 50 μM. The arrow indicates custard apple shaped cells. All images were magnified at 200 x and are representative of at least 3 such fields of view per sample and three independent repeats

The morphology of the control BT-474 cells (Fig. 2a) showed no major changes over the 96 h culture period (except for the increased cell density) with most cells being alive, attached to the substratum and flat in appearance. However, the morphology of the cardanol treated cells (IC_50_ concentration of 15.6 ± 1.76 μg/ml) was different, with unattached cells being observed after 48 h incubation, whilst the attached cells had started to shrink and large clumps of cells were observed at 72 h after treatment with markedly lower cell numbers being visible after 96 h (Fig. 2b).

Doxorubicin treatment (0.5 μg/ml) induced broadly similar changes in the BT-474 morphology as those induced by cardanol, except smaller clumps of custard apple shaped cells and a lower number of viable cells was observed (Fig. 2c).

### Induction of apoptosis and necrosis

**Fig. 3:**
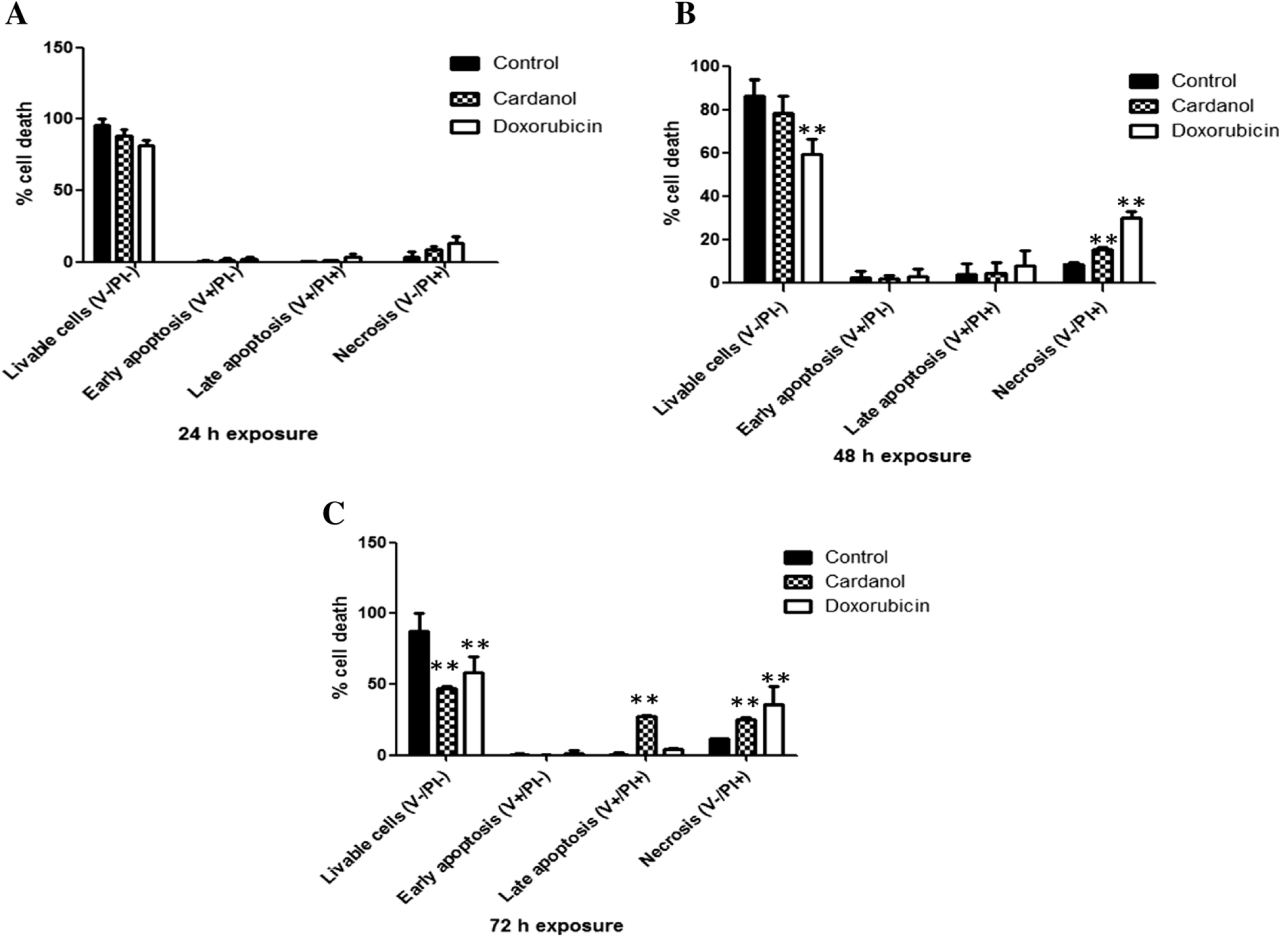
The percentage of cell death. Three groups of cells which were stained untreated cells, cardanol treated cells and doxorubicin treated cells were used. The percentage of livable cells, early apoptosis, late apoptosis, and necrosis was shown. **a**, **b** and **c** represented 24, 48 and 72 h of incubation. A symbol of “**” represented significant difference between the control and treated cells at *p* < 0.01

The induction of apoptosis and necrosis in BT-474 cells was determined by the distribution of annexin V and PI stained cells using flow cytometry. Representative flow cytometry dot plots are shown in Additional file 1: Figure S1 in the supplementary information (SI), whilst the analysis of all three replications is summarized in Fig. 3. The control cells remained largely viable (98 % at 24 h to 78 % at 72 h) with very few apoptotic cells. In contrast, the cardanol (30 μg/ml) treated cells were dead by late apoptosis at 72 h of incubation (27.2 ± 1.1 %), whereas the doxorubicin (0.5 μg/ml) treated cells had mostly died by necrosis from 48 h of incubation (29.9 ± 2.9 %) and this proportion was higher (35.8 ± 13.0 %) after 72 h of incubation. Significant difference between the control and treated cells in both groups could be noticed after 48 h of exposure.

### Cell cycle arrest

The cell cycle position, in terms of the interphase subphases G_1_, S and G_2_/M, were identified by the DNA content as determined by flow cytometric analysis of PI stained cells. Representative histograms (PI fluorescence *vs.* number of cells) are shown in Additional file 2: Figure S2 (SI) and a summary of all the data is shown in Table 2.

**Table 2:** Summary of the percentage of cells in each interphase subphase of the cell cycle

Subphase	Control			Cardanol treated cells			Doxorubicin treated cells		
	24 h	48 h	72 h	24 h	48 h	72 h	24 h	48 h	72 h
Early G_1_	1.1 ± 0.7	1.3 ± 0.9	1.1 ± 1.0	1.7 ± 1.2	2.5 ± 0.6	2.0 ± 1.3	2.0 ± 0.9	3.5 ± 1.1	5.3 ± 3.9
G_1_	66.2 ± 11.4	67.2 ± 6.3	71.5 ± 8.7	72.9 ± 10.2	74.6 ± 3.4	80.7 ± 4.1	59.5 ± 11.5	46.9 ± 3.4	31.8 ± 7.9
S	10.2 ± 1.1	7.7 ± 0.5	7.3 ± 1.7	8.5 ± 4.5	6.5 ± 7.2	5.8 ± 2.2	13.9 ± 1.8	14.4 ± 7.2	14.0 ± 2.2
G_2_/M	19.3 ± 10.2	20.1 ± 6.5	17.0 ± 8.2	14.3 ± 6.7	13.2 ± 8.2	9.2 ± 5.8	20.6 ± 10.4	30.0 ± 8.2	41.3 ± 1.4

Data are shown as the mean ± 1 SD, derived from three independent repeats

For the control cells after 24–72 h culture, around 17–19.3 % of the cells were in the G_2_/M phase and 66–71.5 % in the G_1_ phase of the cell cycle. Cardanol (30 μg/ml) treatment increased the proportion of cells in the G_1_ subphase of the cell cycle compared to the control cells at all three time points, from 66.2 to 72.9 %, 67.2 to 74.6 % and 71.5 to 80.7 % at 24, 48 and 72 h, respectively. Thus, cardanol appeared to induce the cell cycle arrest of BT-474 cells at the G_1_ subphase. Furthermore, 0.5 μg/ml doxorubicin increased the proportion of cells in the G_2_/M subphase of the cell cycle compared to the control at all three time points, from 19.3 to 20.6 %, 20.1 to 30.0 %, and 17.0 to 41.3 % at 24, 48 and 72 h, respectively. Thus, doxorubicin induced cell cycle arrest of BT-474 cells at the G_2_/M subphase.

### Changes in gene transcript expression levels

Since 30 μg/ml cardanol induced the late apoptosis of BT-474 cells after 72 h of incubation, total RNA was extracted from BT-474 cells at this period and the transcript level of genes in the apoptosis and cell cycle regulating groups were evaluated by single stage qRT-PCR.

**Fig. 4:**
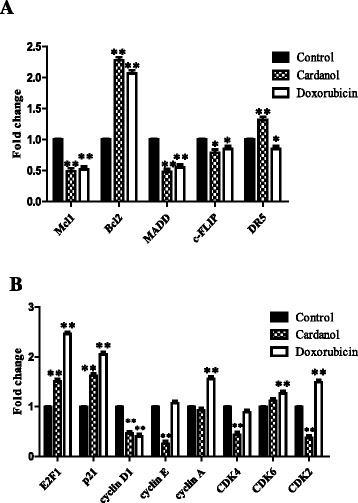
The change in transcript expression levels of genes in the **a** death receptor group (apoptosis regulating) and **b** cell cycle regulating genes (transcription factors important for the cell cycle). BT-474 cells were cultured for 72 h in CM with the addition of DMSO only (Control) or 30 μg/ml cardanol or 0.5 μg/ml doxorubicin. Data are shown as the mean ± 1 SD, derived from three independent repeats. Significant difference between the control and treated cells are shown at the (**) *p* < 0.01 and (*) *p* < 0.05 levels

Within the apoptosis regulating genes evaluated, cardanol (30 μg/ml) treatment increased the transcript expression level of *DR5* and *Bcl-2* significantly at *p* < 0.01 but decreased significantly that of *Mcl-1* (*p* < 0.01), *MADD* (*p* < 0.01) and *c-FLIP* (*p* < 0.05). Doxorubicin (0.5 μg/ml) up-regulated the transcript expression level of *Bcl-2* significantly at *p* < 0.01 but down-regulated significantly that of *Mcl-1* (*p* < 0.01), *MADD* (*p* < 0.01)*, c-FLIP* (*p* < 0.05) and *DR5* (*p* < 0.05) (Fig. 4a). A significant difference at either *p* < 0.01 or *p* < 0.05 levels was compared between the control and treated cells.

For the cell cycle regulation group of genes evaluated, 30 μg/ml cardanol increased the transcript expression level of *p21* and *E2F1* but decreased that of *cyclin D1, cyclin E*, *CDK4* and *CDK2* significantly at *p* < 0.01, whilst 0.5 μg/ml doxorubicin up-regulated significantly *E2F1*, *p21*, *cyclin A*, *CDK6* and *CDK2* transcript expression levels (Fig. 4b). A significant difference at *p* < 0.01 level was compared between the control and treated cells.

### Changes in protein expression levels by western blot analysis

**Fig. 5:**
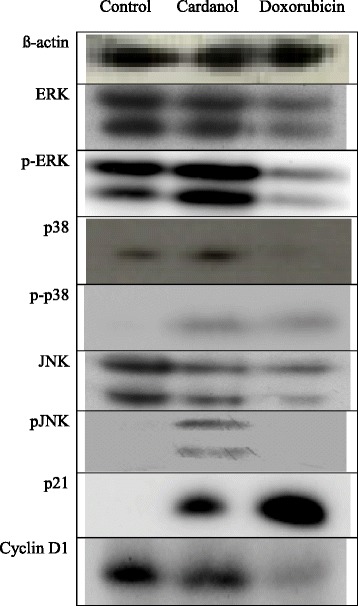
Western blot analysis of protein expression levels in BT-474 cells after incubation in CM with the addition of DMSO only (Control) or with 30 μg/ml cardanol or 0.5 μg/ml doxorubicin for 24 h. Unphosphorylated and phosphorylated forms (p-) of the ERK, p38 and JNK proteins are shown. The blot shown is representative of those seen from 3 independent repeats

The protein expression levels of ERK, JNK and p38 MAPK plus their phosphorylated (active) forms (p-ERK, p-JNK and p-P38), as well as p21 and cyclin D1 in BT-474 cells was evaluated after a 24 h incubation with or without 30 μg/ml cardanol or 0.5 μg/ml doxorubicin (Fig. 5). Cardanol activated ERK, JNK and p38 MAPK, as seen by the increased expression levels of the phosphorylated forms of these three proteins. The increased phosphorylation of ERK, JNK and p38 MAPK, and so their active enzyme levels, is likely to have caused the increased the p21 and cyclin D1 expression levels. Overall, the results strongly suggested that the G_1_ subphase arrest induced by cardanol was mediated by activation of the MAPK-p21 pathway.

## Discussion

Bees collect nectar, bee pollen and resin from different plants and so many compounds can found in bee products. For example, α-pinene was reported to be the major compound in European propolis, and it originated from many plant species, including coniferous species like *Cupressus sempervirens* [21]. Moreover, galangin, chrysin and pinocembrin were found to be the main compounds in Serbian propolis, and were similar to the composition of the resin in poplar trees that are widely distributed in Europe [22]. The propolis from Hungary, Bulgaria, France and Northern Italy were all found to contain resin from poplar trees as well, although the major compounds found in those propolis types were the non-terpenic compounds of benzyl alcohol and benzyl benzoate [23]. Benzyl benzoate was not detected in the volatile oils of poplar buds, although this might reflect differences in the volatiles of different poplar subspecies. Thus, the bud exudates of even of the same species can demonstrate quantitative variability.

Cardanol inhibited the growth of BT-474 cells in a time- and dose-dependent manner (Fig. 1), which is broadly consistent with other reports. For example, the CEE of propolis harvested from many regions in Korea inhibited the angiogenesis, as in tube formation of human umbilical vein endothelial cells (HUVECs), in a dose-dependent manner (6.25–25 μg/ml) [24]. In addition, the CEE of propolis from the Uijeongbu and Pyoseon regions significantly suppressed the proliferation of HUVECs in a dose dependent manner (3.13–25 μg/ml) [24].

Considering the induction of apoptosis by cardanol (Fig. 3 and Additional file 1: Figure S1), cardanol killed BT-474 cells at the late apoptosis (apoptosis and necrosis) stage, which was somewhat similar to doxorubicin, a currently used chemotherapeutic drug, although the later had a higher proportion of necrotic cells. The induction of apoptosis like this is commonly found in compounds purified from natural products, including chemotherapeutic drugs. In addition to propolis, Tualang honey induced late apoptosis in human breast adenocarcinoma (MCF-7 and MDA-MB-231) and cervical (HeLa) cancer cell lines with an EC_50_ value of 2.4–2.8 % (v/v) [25]. MDA-MB-231 cells treated with Tualang honey at 24 h showed the highest percentage of late apoptosis at 37.8 %, while for MCF-7 and Hela cells it was 55.6 and 56.2 %, respectively [25].

The mechanism of induction of apoptotic cells has many pathways. In this study, cardanol increased the transcript expression level of the *Bcl-2* and *DR5* apoptosis-related genes but decreased that of *Mcl-1*, *c-FLIP* and *MADD* (Figs. 4 and 5), somewhat similar to doxorubicin (positive control). The DR5 protein is an apoptosis inducing membrane receptor for TNF-related apoptosis-inducing ligand, where apoptosis in human renal cancer cells is induced by up-regulation of *DR5* and down-regulation of *c-FLIP* [26]. In addition, the combined treatment with rosiglitazone and TNF-α-related apoptosis inducing ligand (TRAIL) could induce apoptosis in renal cancer cells via induction of *Bcl-2* overexpression [26]. Similarly acrolein can effectively sensitize human renal Caki cells to TRAIL-induced apoptosis through down-regulating *Bcl-2* and up-regulating *DR5*, mediated via generation of reactive oxygen species and induction of the C/EBP homologous protein [27]. Thus, lowering the TRAIL resistance or increasing the damage of tumor cells could help in cancer therapy. Moreover, the knockdown of *MADD* and *c-FLIP* reduced the resistance to TRAIL-induced apoptosis in SKOV-3 ovarian cancer cells to 64.2 ± 3.0 % [27].

The Mcl-1 protein is an anti-apoptotic member in the *Bcl-2* family of apoptosis regulating proteins. Benzyl isothiocyanate, an anti-cancer agent, causes G_2_/M cell cycle arrest and apoptosis in human leukemia cell lines via the down-regulation of *Mcl-1* [28].

Cardanol appears to arrest BT-474 cells at the G_1_ subphase of the cell cycle, somewhat similar to the effect of propolin H from Taiwanese propolis that arrested the human lung carcinoma H460 cell line in the G_1_ subphase. Treatment of H460 cells with 40 μM of propolin H increased the proportion of cells in the G_1_ subphase from 57.8 to 75.1 % [29].

Cancer cells display an uncontrolled growth and present abnormal gene expression profiles. The expression level of regulating genes, such as cyclin and CDKs, are typically higher and so induce the cell cycle to move to the next phase. With respect to the effect of cardanol on BT-474 cells, it affected the expression of many genes important for the cell cycle, such as decreasing p21, cyclin D1, cyclin E, CDK2 and CDK4 expression levels and increasing that for p21 and E2F1. These results are in accord with those for CAPE at a concentration of 2.5–80 mg/l that increased the proportion of cells in the G_1_ subphase in a dose-dependent manner, and also increased the expression of beta-catenin and decreased the expression of cyclin D1 and c-myc [30]. In addition, a 24 h exposure to CAPE (50 μg/ml) inhibited the growth of C6 glioma cells, inducing cell cycle arrest at the G_1_ subphase after a 24 h incubation, decreasing the CDK2/cyclin E and CDK4/cyclin D activity and inhibiting Rb phosphorylation by increasing p21, p27 and p16 expression [31].

The potential induction of the G_1_ cell cycle arrest by cardanol via increasing p21, p-p38 MAPK, p-JNK and p-ERK protein levels was similar to isothiocyanate sulforaphane, a chemotherapeutic drug. The mechanism of SFN on human colon carcinoma HT-29 cells was reported to be mediated via inducing expression of p21^CIP1^ and cyclin D1 through activating the MAPK pathways, including ERK, JNK and p38 [32].

In this report, doxorubicin was used as positive control since its action is already well reported [33]. It is an anthracyline drug extracted from *Streptomyces peucetius* var *caesivs* and has been used for treatment of diverse cancers, including breast, lung, gastric, ovarian, thyroid, non-Hodgkin’s and Hodgkin’s lymphoma. The mechanism of action of this drug on cancer inhibition has been described in two pathways. First, it binds to DNA and disrupts topoisomerase II-mediated DNA repair. Second, it produces free radicals and damages the cell membrane, DNA and protein leading to cell death. The data of this research supported the effect of doxorubicin on the BT-474 cell line with a cell cycle arrest at the G_2_/M subphase, up-regulated transcript expression levels of *Bcl-2*, *E2F1, p21, cyclin A*, *CDK6* and *CDK2* and down-regulated expression of *Mcl-1, MADD, c-FLIP* and *cyclin D1.* Thus, doxorubicin is likely to act via inhibiting DNA synthesis through increased p21 and cyclin D1 activities. Moreover, doxorubicin decreased the expression level of the anti-apoptotic genes *Mcl-1* and *c-FLIP*.

**Fig. 6:**
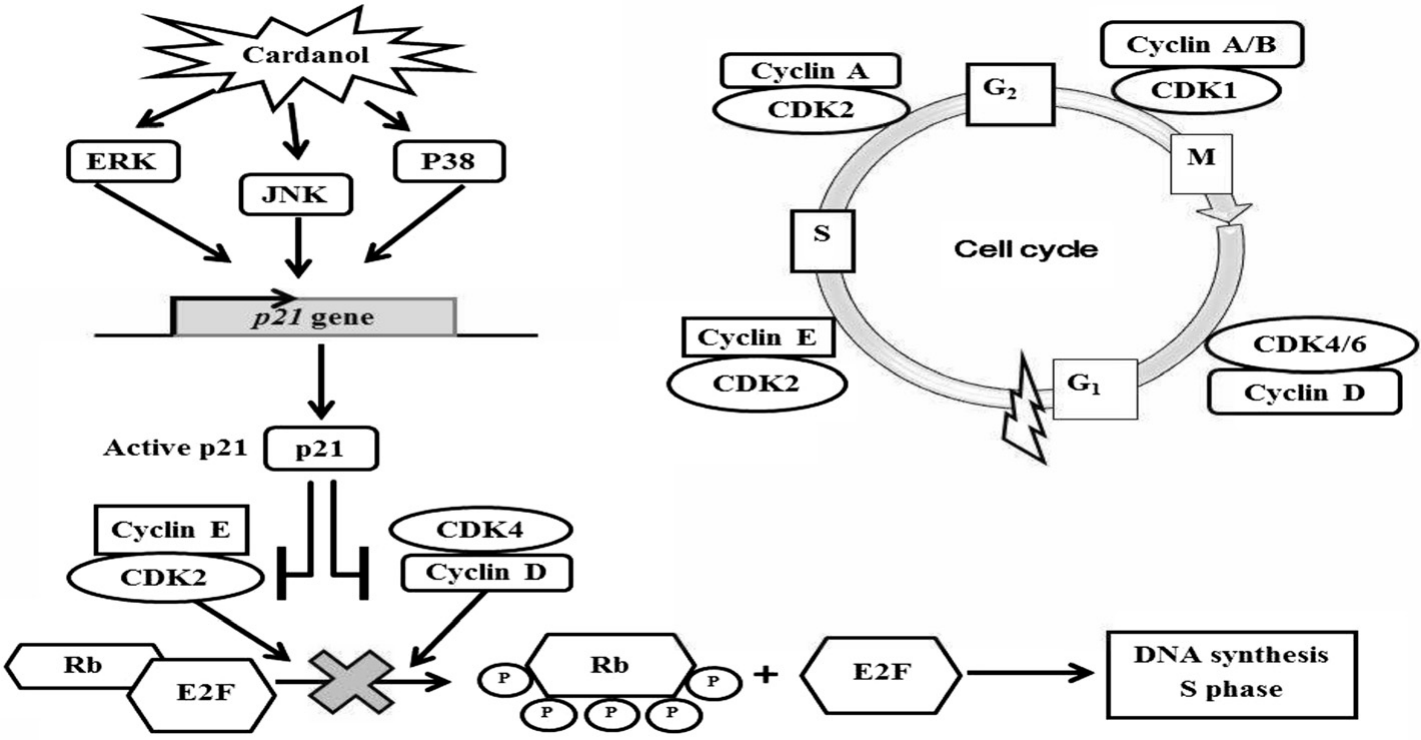
A model of the mechanism of action of cardanol to induce cell cycle arrest at the G_1_ subphase and cell death in BT-474 cancer cells

In summary, the proposed mechanism of how cardanol could inhibit the growth of BT-474 cells is shown in Fig. 6. In this model, cardanol increases the phosphorylation of ERK, JNK and p38 MAPK leading to p21 activation. Then, the active p21 suppressed CDK4/cyclin D and cyclin E/CDK2 and so prevented the hyperphosphorylation of the retinoblastoma protein. This led to the obstruction of DNA synthesis and prevented the movement of cells into and from the S subphase, causing the G_1_ subphase arrest.

However, the data mentioned above were from in vitro only. In the future, primary normal cell culture and animal models must be performed before going forward to human testing. As known, in vitro cultured cells can not represent the whole organism due to lack of precise control of physicochemical surrounding, physiological conditions and so on [34].

In addition, it should be aware of an agent with both anticancer and antioxidant activities. Recently, it has been contradictorily reported whether such compound could be applied to the treatment of cancer [35].

## Conclusion

Cardanol, purified from *Apis mellifera* propolis from Nan province, Thailand, had a cytotoxic activity (IC_50_ value of 15.6 ± 1.76 μg/ml) against the BT-474 cell line. The inhibition by cardanol was time- and dose-dependent manner. Morphologically, cardanol treated cells revealed a loss of adhesion and cell shrinking with the formation of large clumps and a reduced number of viable cells. After 72 h, significant numbers of cells were dead by late apoptosis. The change in transcript and protein expression levels of genes involved in apoptosis induction and cell proliferation strongly suggested that the MAPK regulated p21-mediated G_1_ phase cell cycle arrest was a mechanism underlying the growth inhibitory effect of cardanol on BT-474 cells.

## Additional files

Additional file 1:
**Program cell death of BT-474 cells.** A, B and C represented untreated cells as control, 30 μg/ml cardanol treated cells and 0.5 μg/ml doxorubicin treated cells while I, II and III represented 24, 48 and 72 h of incubation, respectively. Duplication of experiments was done. This figure was from one replication only. (DOCX 91 kb) Additional file 2:
**The cell cycle arrest of BT-474 cells.** (A) Control, (B) 30 μg/ml cardanol treated and (C) 0.5 μg/ml doxorubicin treated cells after (I) 24 h, (II) 48 h and (III) 72 h of incubation. Histograms shown are derived from 2 events (cells) and are representative of three independent repeats. (DOCX 235 kb)
