# Supplementary figures and images for: Cardanol isolated from Thai Apis mellifera propolis induces cell cycle arrest and apoptosis of BT-474 breast cancer cells via p21 upregulation
Source: Daru. 2015 Dec 22;23:55. doi: 10.1186/s40199-015-0138-1 (PMC4687141; doi:10.1186/s40199-015-0138-1)

**
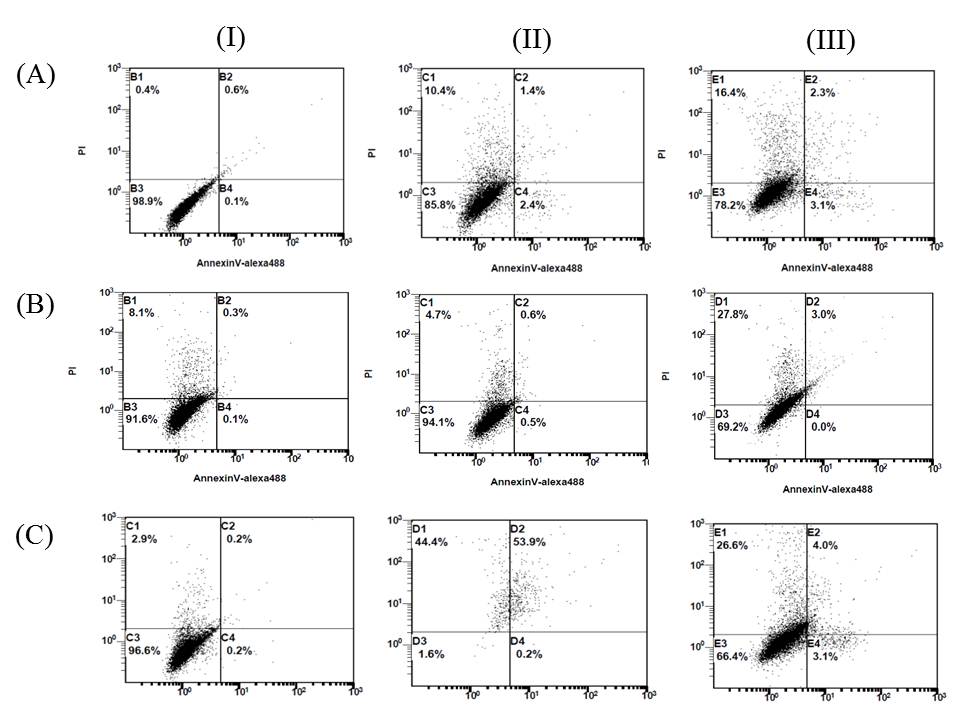
**

Additional file 1

Supplement: Additional file 1: — Program cell death of BT-474 cells. A, B and C represented untreated cells as control, 30 μg/ml cardanol treated cells and 0.5 μg/ml doxorubicin treated cells while I, II and III represented 24, 48 and 72 h of incubation, respectively. Duplication of experiments was done. This figure was from one replication only. (DOCX 91 kb) [file 40199_2015_138_MOESM1_ESM.docx]

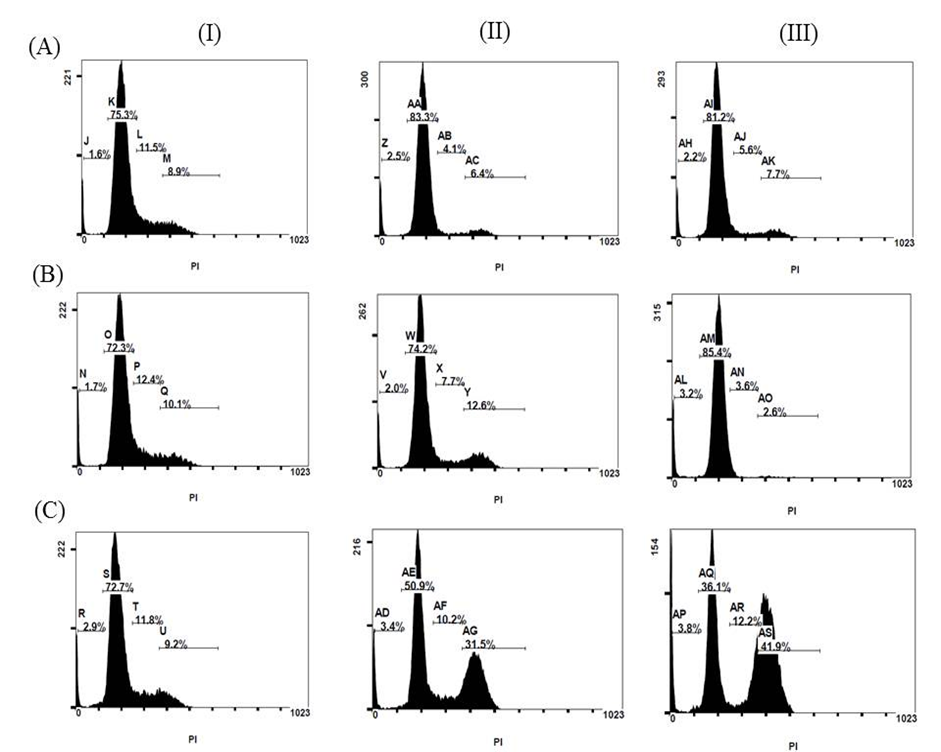


Additional file 2

Supplement: Additional file 2: — The cell cycle arrest of BT-474 cells. (A) Control, (B) 30 μg/ml cardanol treated and (C) 0.5 μg/ml doxorubicin treated cells after (I) 24 h, (II) 48 h and (III) 72 h of incubation. Histograms shown are derived from 2 events (cells) and are representative of three independent repeats. (DOCX 235 kb) [file 40199_2015_138_MOESM2_ESM.docx]
